# PET/CT imaging 2 h after injection of [^18^F]PSMA-1007 can lead to higher staging of prostate cancer than imaging after 1 h

**DOI:** 10.1186/s41824-023-00167-4

**Published:** 2023-05-01

**Authors:** Erland Hvittfeldt, Ulrika Bitzén, David Minarik, Jenny Oddstig, Berit Olsson, Elin Trägårdh

**Affiliations:** 1 Department of Translational Medicine, Wallenberg Centre for Molecular Medicine, Lund University, Carl Bertil Laurells gata 9, 205 02 Malmö, Sweden; 2 Department of Clinical Physiology and Nuclear Medicine, Skåne University Hospital, Malmö, Sweden; 3 Department of Clinical Physiology and Nuclear Medicine, Skåne University Hospital, Lund, Sweden; 4 Department of Medical Physics, Skåne University Hospital, Malmö, Sweden; 5 Department of Medical Physics, Skåne University Hospital, Lund, Sweden

**Keywords:** [^18^F]PSMA-1007, Prostate cancer, Uptake time, PET/CT, PSMA

## Abstract

**Background:**

[^18^F]PSMA-1007 is a prostate specific membrane antigen (PSMA) ligand for positron emission tomography (PET) imaging of prostate cancer. Current guidelines recommend imaging 90–120 min after injection but strong data about optimal timing is lacking. Our aim was to study whether imaging after 1 h and 2 h leads to a different number of detected lesions, with a specific focus on lesions that might lead to a change in treatment.

**Methods:**

195 patients underwent PET with computed tomography imaging 1 and 2 h after injection of [^18^F]PSMA-1007. Three readers assessed the status of the prostate or prostate bed and suspected metastases. We analyzed the location and number of found metastases to determine N- and M-stage of patients. We also analyzed standardized uptake values (SUV) in lesions and in normal tissue.

**Results:**

Significantly more pelvic lymph nodes and bone metastases were found and higher N- and M-stages were seen after 2 h. In twelve patients (6.1%) two or three readers agreed on a higher N- or M-stage after 2 h. Conversely, in two patients (1.0%), two readers agreed on a higher stage at 1 h. SUVs in suspected malignant lesions and in normal tissues were higher at 2 h, but lower in the blood pool and urinary bladder.

**Conclusions:**

Imaging at 2 h after injection of [^18^F]PSMA-1007 leads to more suspected metastases found than after 1 h, with higher staging in some patients and possible effect on patient treatment.

**Supplementary Information:**

The online version contains supplementary material available at 10.1186/s41824-023-00167-4.

## Background

[^18^F]PSMA-1007 is one of several ^18^F-labeled prostate specific membrane antigen (PSMA) ligands available as a radiotracer for use in positron emission tomography/computed tomography (PET/CT) imaging of prostate cancer (Czarniecki et al. 2018). One of its advantages is hepatobiliary (rather than urinary) excretion, leading to lower activity in the urinary bladder which may increase visibility of lesions in this area (Giesel et al. 2017). A disadvantage with this compound is a relatively high number of unspecific lesions, especially problematic in bone (Grunig et al. 2021; Rauscher et al. 2020).

Optimal uptake time of [^18^F]PSMA-1007 before imaging has not been established. A study of 40 patients comparing 1 and 2 h uptake time reported increased activity in suspected malignant lesions, and lower background activity, but only one additional possible lymph node metastasis at 2 h compared with 1 h (Rahbar et al. 2018). Clinical studies of [^18^F]PSMA-1007 have generally used an uptake time of 90–120 min, and this interval is recommended in the recently published EANM/SNMMI guideline for prostate cancer imaging (Giesel et al. 2019; Ingvar et al. 2022; Sprute et al. 2021; Fendler et al. 2023). Imaging after 1 h would allow increased production and higher patient comfort compared to a longer interval.

Our primary aim was to study whether imaging after 1 h and 2 h leads to a different number of suspected metastases, with a specific focus on metastases that might lead to a change in treatment.

## Methods

### Subjects

Data were collected between May 2020 and June 2021. All patients referred to the two nuclear medicine units of Skåne University Hospital (located in Malmö and Lund, respectively) for [^18^F]PSMA-1007 PET/CT were eligible for inclusion. Unless deemed unsuitable for multiple scans (e.g., because of infirmity), patients were offered participation when the clinical schedule allowed for additional scanning. Our departments mainly accept the indications of biochemical recurrence (BCR) after curative treatment and primary staging (PS) of high-risk prostate cancer (modified D’Amico criteria from Swedish guidelines–stage T3–T4 *or* Gleason score 8–10 or 4 + 3 in targeted biopsy of PI-RADS 5 or > 50% of systematic biopsies *or* PSA > 20).

### Imaging

Subjects were injected with 4.0 MBq/kg of [^18^F]PSMA-1007. Four GE Discovery MI PET‐CT systems (GE Healthcare) were used for the examinations. PET/CT scans were performed 1 and 2 h after injection. Images were acquired from the base of the skull to the mid-thighs with an acquisition time of 120 s per bed position at both scan times. At 1 h a low dose CT (120 kV/30–160 Ma, mean DTP 233 mGy*cm) was performed and at 2 h a diagnostic CT (100 kV/80–480 mA, mean DTP 1710 mGy*cm), with intravenous contrast unless contraindicated. A block-sequential regularization expectation maximization algorithm (Q.Clear; GE Healthcare, Milwaukee, WI, USA) was used for reconstruction of PET data, with the noise‐regularization parameter *β* set to 800 (Tragardh et al. 2020). Time-of-flight, point spread function modelling, and a 256 × 256 matrix (with a pixel size of 2.7 × 2.7 mm^2^ and a slice thickness of 2.8 mm) were used.

### Image review

Images were reviewed blinded and independently by two nuclear medicine specialists (ET and UB) and one nuclear medicine resident (EH) with > 3 years’ experience of interpreting PSMA PET/CT scans. The readers judged whether pathologic uptake in the prostate suggestive of cancer was present and if seminal vesicle (SV) involvement was suspected or, alternatively, if local recurrence in the prostate bed was suspected. Readers counted the number of suspected metastases in seven anatomical regions—pelvic lymph nodes (LNs) below the aortic bifurcation, abdominal LNs, LNs cranial to the diaphragm, inguinal LNs and lesions in bone, liver, or lungs. Suspected metastases in other sites and other findings deemed significant were also noted. A maximum of 5 metastases in any region were counted, to save time and to avoid incorrect reporting due to a high number of metastases.

The readers mainly used their clinical experience and judgement to interpret the images. In general, this meant that low-grade (clearly below spleen) symmetrical uptake in lymph nodes in the inguinal regions, axilla, mediastinum or along the distal external iliac arteries was not considered metastases. To minimize the problem of unspecific uptake in bone, readers were instructed to only classify distinct high-grade (at least above spleen) uptake as metastases, and lower uptake only in the presence of possibly malignant sclerotic lesions on CT.

Patients were then classified as N1, M1a, M1b and M1c (for regional lymph nodes, extra-pelvic lymph nodes, bone metastases and other sites, respectively) positive according to the PROMISE criteria (Eiber et al. 2018).

### SUV measurements

A nuclear medicine technologist (BO) placed volumes of interest (VOIs) in the parotid gland, liver, spleen, aorta, and urinary bladder for measurement of SUVmean. SUVmax was measured in lesions which at least one reader judged pathological. For this purpose, EH placed VOIs around the highest uptake in the prostate or prostate bed and a maximum of 2 suspected metastases per anatomical region.

### Statistics

Comparisons were made between findings at 1 and 2 h. Binary outcomes (visibility of primary tumor, SV involvement, local recurrence, and N/M-stages) were compared with McNemar’s test. SUVs and number of suspected metastases found were compared with the Wilcoxon signed-rank test. To compensate for multiple analyses Bonferroni correction was applied. The significance level was set to *p* < 0.005 both for reader findings (11 comparisons) and SUVs (10 comparisons). Analyses were made with IBM^®^ SPSS^®^ Statistics 28.0.0.0.

## Results

### Subjects

197 patients were recruited. Two patients were excluded: one due to unsuitable indication (follow-up of ^177^Lu therapy in widespread metastatic disease) and one due to technical problems with images at 1 h. Thus, a total of 195 patients were included, all with histopathologically verified prostate cancer. Patient data are reported in Table 1.

**Table 1:** Patient data

	All subjects (*n* = 195)					
Age*	70 (53–83)					
Injected dose (MBq/kg)**	4.0 ± 0.2 (2.7–6.0)					
ISUP grade	1	2	3	4	5	?
(*n*)	4	39	46	38	59	9
Scan time after injection (min)**	Scan 1	59.8 ± 1.8 (55–69)		Scan 2	120.2 ± 3.6 (115–142)	
Indication	Primary staging (*n* = 128)			BCR (*n* = 67)		
PSA at scanning (µg/l)*	11 (0.85–579)			4.1 (0.17–62)		
Previous prostatectomy (*n*)	–			56		
Previous external radiation (*n*)	–			8		
Previous brachy-/cryotherapy (*n*)	–			3		
Hormone treatment at scan (*n*)	–			10		

*Median (range) **mean ± SD (range)

### Image review

**Fig. 1:**
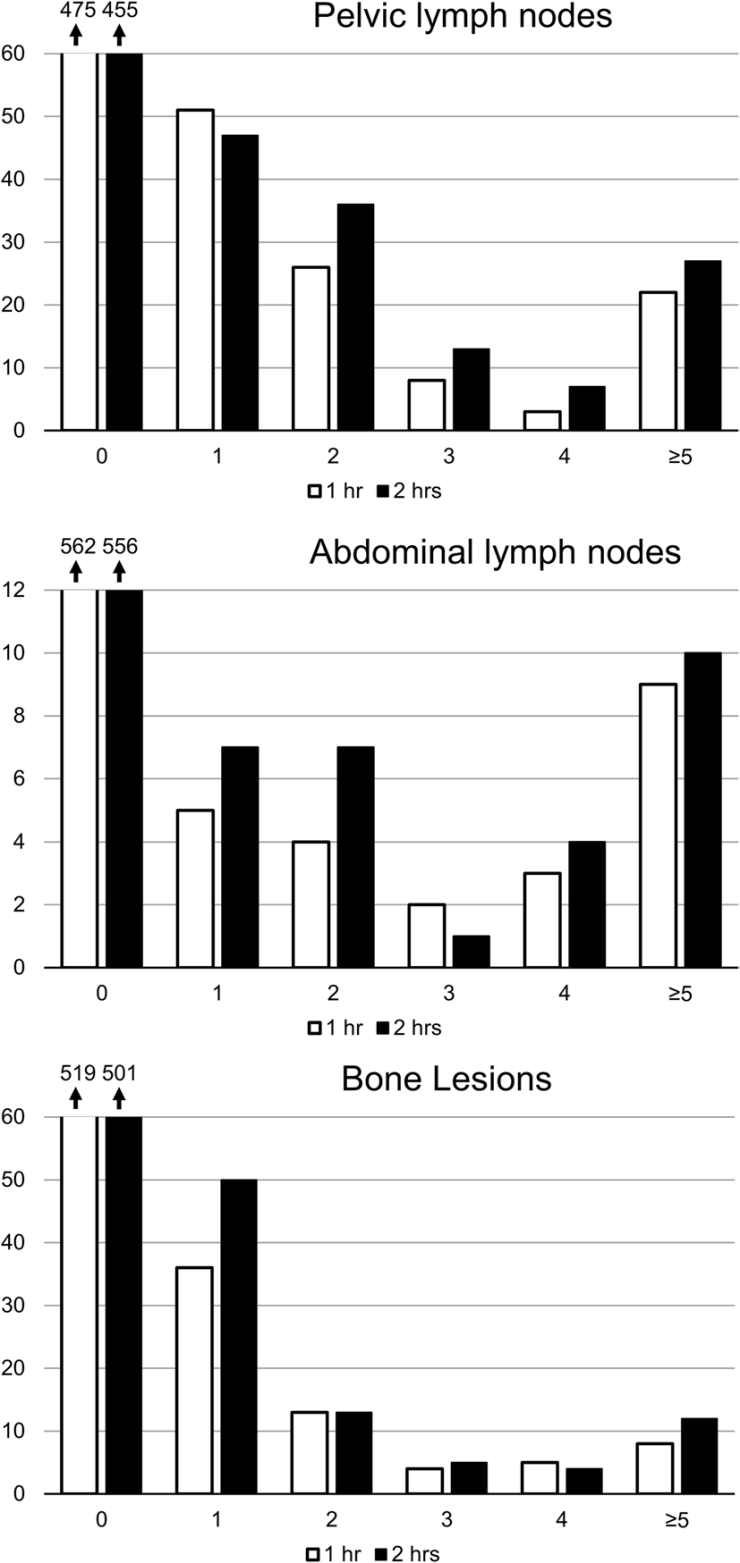
Distribution of number of found suspected metastases (*x*-axis) at 1 and 2 h in three anatomical regions, in 3 readings of 195 patients (*n* = 3 × 195 = 585)

We analyzed the difference between findings at 1 and 2 h in 585 separate readings (3 readers and 195 subjects). The main findings are summarized in Table 2. More suspected metastases were found at 2 h as compared to 1 h in all lymph node regions and in bone. In primary staging, pathological uptake in the prostate was more often seen and SV involvement was more often suspected at 2 h. In BCR, local recurrence was more often suspected at 2 h. N and M stages were higher at 2 h. Statistical significance was seen for pelvic LNs, bone metastases, SV involvement and all N/M-stages (except M1c which was not analyzed, see below). Distribution of number of suspected metastases found at 1 and 2 h for pelvic LNs, abdominal LNs and in bone are shown in Fig. 1.

**Table 2:** Findings by individual readers

	Any metastases found		Change in number of found metastases		
	At 1 h	At 2 h	More at 1 h	More at 2 h	*p*^c^
Pelvic LNs *n* (%)	110 (18.8%)	130 (22.2%)	7 (1.2%)	51 (8.7%)	< 0.001*
Abdominal LNs	23 (3.9%)	29 (5.0%)	1 (0.2%)	10 (1.7%)	0.008
Supradiafragmal LNs	19 (3.2%)	20 (3.4%)	0	5 (0.9%)	0.039
Inguinal LNs	7 (1.2%)	11 (1.8%)	0	5 (0.9%)	0.039
Bone	66 (11.3%)	84 (14.4%)	10 (1.7%)	30 (5.1%)	0.003*

	Status of prostate/prostate bed				
	At 1 h	At 2 h	Only at 1 h	Only at 2 h	*p*^d^
Primary tumor detected^a^	342 (89.1%)	354 (92.2%)	7 (1.8%)	19 (4.9%)	0.031
SV involvement suspected^a^	67 (17.4%)	83 (21.6%)	4 (1.0%)	20 (5.2%)	0.002*
Local recurrence suspected^b^	48 (23.9%)	56 (27.9%)	1 (0.5%)	9 (4.5%)	0.021

	N- or M-stage				
	At 1 h	At 2 h	Higher at 1 h	Higher at 2 h	*p*^d^
N1 disease	110 (18.8%)	130 (22.2%)	4 (0.7%)	24 (4.1%)	< 0.001*
M1a disease	31 (5.3%)	41 (7.0%)	0	10 (1.7%)	0.002*
M1b disease	66 (11.3%)	84 (14.4%)	4 (0.7%)	22 (3.8%)	< 0.001*

*n* = 585 (3 readings of 195 patients). *n* indicates number of readings, not number of suspected metastases

^a^Primary staging only (*n* = 3 × 128 = 384)

^b^BCR only (n = 3 × 67 = 201)

^c^Using Wilcoxon signed rank test to test for difference in number of found metastases at 1 and 2 h

^d^Using McNemar’s test to test for difference in status/stage at 1 and 2 h

*p < 0.005

M1c disease was uncommon, and no statistics were performed. Suspected lung metastases were found in one patient, with > 5 metastases reported by all readers at both scan times. In one patient, suspected metastases to the abdominal wall was detected by all readers at both time points. In ten patients, uptakes deemed unrelated to prostate cancer were found. These findings are reported in Additional file 1: Table S1. Two of these were focal uptakes in liver, both in the primary staging setting with no other metastases reported, so liver metastases were considered unlikely.

**Fig. 2:**
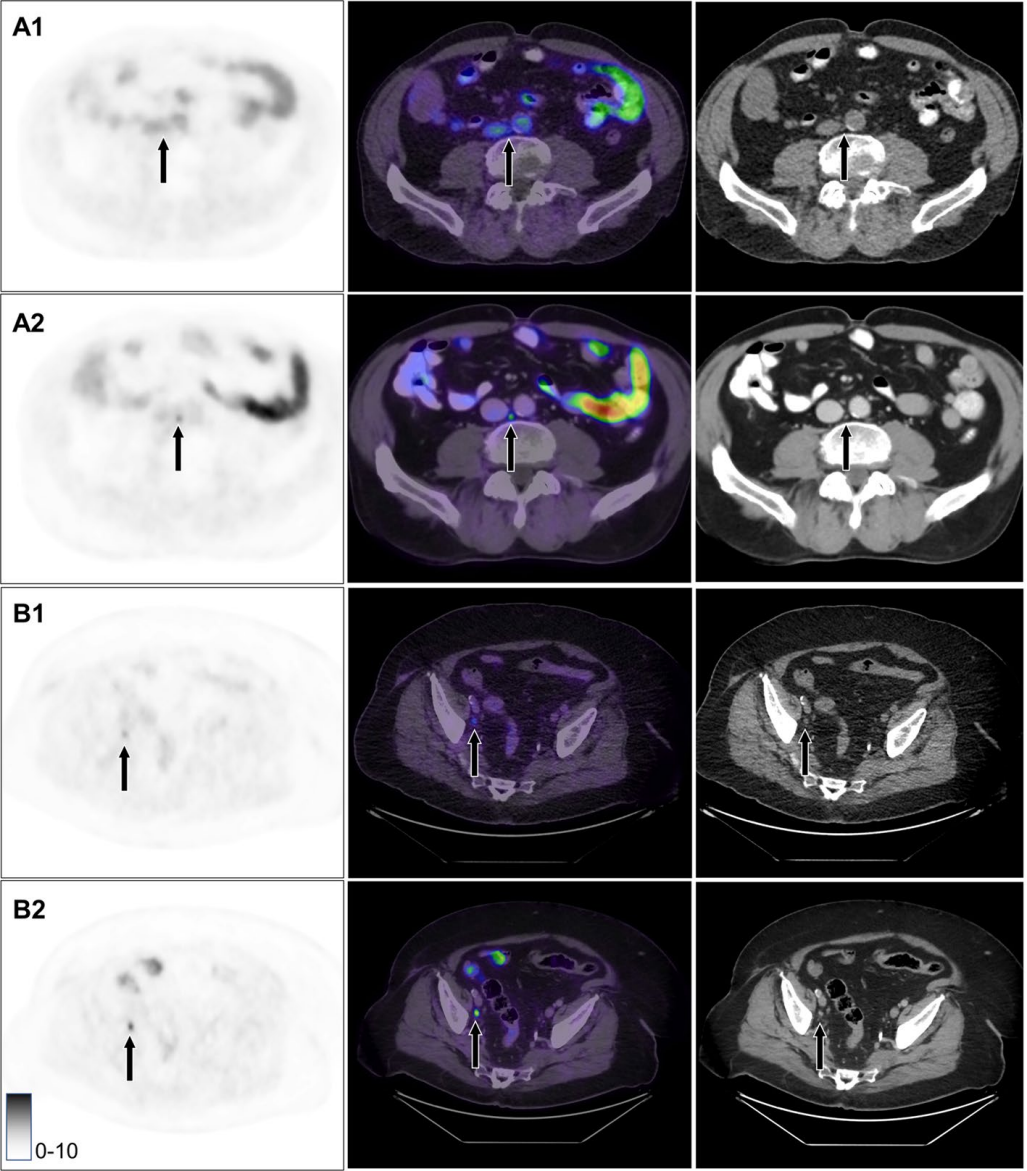
Suspected metastases which were not found by any reader after 1 h but by all readers after 2 h. **A1** Paraaortic lymph node, imaging at 1 h. **A2** Same lymph node at 2 h. One more paraaortic lymph node was found in the same patient only at 2 h. **B1** Pelvic lymph node at 1 h. **B2** Same lymph node at 2 h

In two cases (1.0% of all cases) all three readers agreed on a higher N- or M-stage at 2 h (Fig. 2), while in ten cases (5.1%) two readers agreed on higher N- or M-stage at 2 h. Conversely, in two cases (1.0%) two readers agreed on a higher N- or M-stage at 1 h. Cases of agreement between two or three readers are listed in Table 3.

**Table 3:** Patients where at least two readers agreed on a different assessment at 1 and 2 h

Reader agreement	Higher stage at 1 h		Higher stage at 2 h	
	2 readers	3 readers	2 readers	3 readers
Primary tumor visible*	0	1	4	1
Vesicular involvement*	0	0	3	0
Local recurrence**	0	0	3	0
N1 disease	1	0	7	1
M1a disease	0	0	1	1
M1b disease	1	0	2	0

(*n* = 195)

*Primary staging only (*n* = 128). **BCR only (*n* = 67)

### SUV measurements

Measured SUV was higher after 2 h in suspected malignant lesions and in normal tissues with statistical significance except for lesions in the prostate bed. Measured SUV was significantly lower after 2 h in the aorta and the urinary bladder. See Table 4.

**Table 4:** SUV at 1 and 2 h in suspected malignant lesions and normal organs

Suspected malignant lesions	SUVmax at 1 h	SUVmax at 2 h	*p*
Bone (*n* = 177)	5.1 (4.0–8.6)	7.5 (5.6–12.0)	< 0.001*
Prostate (*n* = 118)	13.3 (8.5–23.0)	17.2 (11.2–34.1)	< 0.001*
Prostate bed (*n* = 18)	8.4 (4.6–14.2)	12.3(5.8–17.9)	0.021
Lymph nodes (*n* = 116)	5.7 (3.3–12.9)	9.5 (5.4–20.8)	< 0.001*

Normal organs (*n* = 195)	SUVmean at 1 h	SUVmean at 2 h	
Aorta	2.1 (1.7–2.4)	1.0 (0.8–1.3)	< 0.001*
Bladder	3.2 (1.7–4.8)	1.2 (0.7–2.1)	< 0.001*
Liver	10.5 (8.8–12.8)	12.9 (10.9–15.3)	< 0.001*
Parotid	3.6 (3.0–4.4)	4.0 (3.2–4.8)	< 0.001*
Spleen	9.6 (7.7–12.0)	11.5 (8.7–14.8)	< 0.001*

SUVs are Median (interquartile range)

*p < 0.005

## Discussion

In this study, we compared PET/CT imaging of 195 prostate cancer patients 1 and 2 h after injection of [^18^F]PSMA-1007 and compared the number of suspected malignant lesions found and whether this changed the N- and M-stage of the disease. In general, more lesions were found after 2 h, with higher N- and M-stages as a result. There was inter-reader variability (which we did not analyze statistically) but in twelve patients (6.1%) two or three of our readers agreed on a higher N- or M-stage (most often due to additional lymph nodes found), with likely effect on patient treatment.

Our results are in line with the previous study by Rahbar et al., which compared imaging at 1 and 2 h after injection [^18^F]PSMA-1007 in 40 patients. They reported higher uptake in suspected malignant lesions at 2 h, but only one possible additional lymph node detected (Rahbar et al. 2018). The main advantage of our study is the higher number of patients and reviewers, and the focus on staging. Giesel et al. reported higher uptake in suspected malignant lesions at 3 h compared to 1 h, but no difference in the number of lesions, in 12 patients with BCR (Giesel et al. 2018). Lau et al. reported no difference in the number of found extra-prostatic lesions in 68 patients scanned at a mean 94 and 144 min after injection (Lau et al. 2022). The focus of their study was delineation of lesions, so the results cannot readily be used for determining optimal uptake time.

There are some limitations of this study to address. We did not seek histopathological verification of any of the metastases detected on PET/CT. Several studies with histopathological verification have shown high specificity of PSMA ligands for lymph node metastases, including one from our own center performed with [^18^F]PSMA-1007 (Ingvar et al. 2022; Baas et al. 2022; Hope et al. 2021; Pienta et al. 2021). Accuracy of PSMA PET for bone lesions with histopathological verification is less studied and considering the known problem of unspecific bone uptakes with [^18^F]PSMA-1007, extrapolating from studies with other PSMA ligands could be misleading. It is likely that some number of the increase in suspected bone metastases is due to unspecific bone uptake. Research regarding PSMA PET accuracy for SV involvement is also limited with varied results (Prive et al. 2021; Sonni et al. 2022; Chen et al. 2020). We believe that as the main findings in our study concern lymph nodes (ten of twelve cases where at least two readers agreed on a higher stage were due to additional lymph nodes found), where specificity is high, our conclusions should not be greatly affected by the lack of verification.

We used two different CT protocols—a diagnostic CT at our standard imaging time of 2 h and a low dose CT at 1 h to limit radiation dose to the patient—which leads to two problems. First, blinding is affected as reviewers can deduct the time after injection from the CT images. This increases the risk of bias, but the magnitude and direction of this possible bias is hard to evaluate. Second, the interpretation of the PET scan can be affected by CT findings. For example, the morphology of lymph nodes, the visibility of subtle bone lesions and the delineation of the seminal vesicles could differ with different protocols. Again, the effect is hard to evaluate but is likely small. A diagnostic CT is not considered obligatory in PSMA PET imaging (Fendler et al. 2023).

A possible point of contention is our use of the aortic bifurcation as the classifier for M1a disease, in line with the PROMISE criteria and E-PSMA guidelines but contrary to the 8th edition of the TNM criteria which uses the iliac bifurcation (Fendler et al. 2023; Brierley et al. 2017; Ceci et al. 2021). There is ongoing debate about which is the clinically relevant M1a classifier (Arnfield et al. 2022; Oprea-Lager et al. 2022). In a 2020 Dutch multidisciplinary consensus meeting 39% of panelists chose the iliac bifurcation while 22% chose the aortic bifurcation (Mason et al. 2022). We chose the aortic bifurcation because it is used by our colleagues in the local urology department. Our use of classifier might affect the precise number of reclassified patients but not the fact that more lymph nodes were found at the different time points.

In summary, our data suggests that 2 h uptake time is preferable to 1 h, at least in primary staging and BCR. However, the results are not immediately generalizable to all clinical scenarios—for example we scanned no patients with castration resistant metastatic prostate cancer. There may be other situations when 1 h uptake time is acceptable, for example when it is not important to find additional small lesions (such as follow-up of patients with known high tumor burden).

## Conclusions

This study demonstrates that [^18^F]PSMA-1007 PET scans at different time points after injection lead to a difference in the number of found suspected prostate cancer lesions, with generally more lesions being found after 2 h as compared to 1 h. In some patients this can lead to a change in staging with possible consequences for treatment and patient outcome.

## Supplementary Information

**Additional file 1**. List of patients with reported findings likely unrelated to prostate cancer — site and number of findings.

## Data Availability

The datasets used during the current study are available from the corresponding author on reasonable request.

## Abbreviations

BCR: Biochemical recurrence
CT: Computed tomography
LN: Lymph node
PET: Position emission tomography
PS: Primary staging
PSMA: Prostate specific membrane antigen
SUV: Standardized uptake value
SV: Seminal vesicles
VOI: Volume of interest
