# Supplementary material for: PET/CT imaging 2 h after injection of [18F]PSMA-1007 can lead to higher staging of prostate cancer than imaging after 1 h
Source: Eur J Hybrid Imaging. 2023 May 1;7:9. doi: 10.1186/s41824-023-00167-4 (PMC10149540; doi:10.1186/s41824-023-00167-4)
Supplement: Supplementary file 1 — Additional file 1. List of patients with reported findings likely unrelated to prostate cancer — site and number of findings. [file 41824_2023_167_MOESM1_ESM.pdf]

	Reader 1		Reader 2		Reader 3	
	1 hour	2 hours	1 hour	2 hours	1 hour	2 hours
Patient 1<br>Focal liver uptake[a]	1	1	1	—	1	1
Patient 2<br>Focal liver uptake[a]	2	—	—	2	2	2
Patient 3<br>Lung cancer? [a]	1	1	1	—	1	1
Patient 4<br>Lung cancer?	1	1	—	—	1	1
Patient 5<br>Lung cancer?	1	1	1	1	1	1
Patient 6<br>Ureteral cancer?	1	1	1	1	—	—
Patient 7<br>Focal uptake in gastric ventricle	1	1	—	—	1	1
Patient 8<br>Focal thyroid uptake	—	—	—	—	1	1
Patient 9<br>Focal thyroid uptake	—	—	—	—	1	1
Patient 10<br>Focal uptake in spleen	3	3	—	—	5	5

Supplementary table 1. List of patients with reported findings likely unrelated to prostate cancer — site and number of findings. [a] indicates any intra-reader difference between time points.
